# Cohort Profile: The 10/66 study

**DOI:** 10.1093/ije/dyw056

**Published:** 2016-05-05

**Authors:** A. Matthew Prina, Daisy Acosta, Isaac Acosta, Mariella Guerra, Yueqin Huang, A.T. Jotheeswaran, Ivonne Z. Jimenez-Velazquez, Zhaorui Liu, Juan J. Llibre Rodriguez, Aquiles Salas, Ana Luisa Sosa, Joseph D. Williams, Martin Prince

**Affiliations:** 1 King’s College London, Centre for Global Mental Health, Institute of Psychiatry, Psychology & Neuroscience, London, UK,; 2 Universidad Nacional Pedro Henriquez Ureña, Internal Medicine Department, GeriatricSection, Santo Domingo, Dominican Republic,; 3 National Institute of Neurology and Neurosurgery of Mexico, National Autonomous University of Mexico, Mexico City, Mexico,; 4 Instituto de la Memoria y Desordenes Relacionados, Lima, Perú,; 5 Peking University, Institute of Mental Health, Beijing, China,; 6 Department of Ageing and Life Course, World Health Organization, Geneva,; 7 Internal Medicine Department, Geriatrics Program, Medical Sciences Campus, University of Puerto Rico, San Juan, Puerto Rico,; 8 Facultad de Medicina Finlay-Albarran, Medical University of Havana, Havana, Cuba,; 9 Medicine Department, Caracas University Hospital, Universidad Central de Venezuela, Caracas, Venezuela and; 10 Department of Community Health, Voluntary Health Services, Chennai, India

## Why was the cohort set up?

Population ageing is affecting low- and middle-income countries, with absolute and relative numbers of older adults increasing quickly across the globe. This demographic transition is accompanied by a health transition, driven by changing habits and lifestyles, where non-communicable diseases are becoming the major cause of morbidity. Dementia is strongly associated with age and is one of the main contributors to dependence and disability. It has been estimated that there are nearly 47 million people currently living with dementia, most of whom live in low- and middle-income countries (LMICs).

The title of the 10/66 Dementia Research Group (DRG) reflects the fact that, when the group was formed in 1998, less than 10% of population-based research on dementia had been carried out in developing countries although two-thirds of those affected lived in those settings.^1^ The 10/66 DRG research programme was developed to address this inequity, quantifying dementia prevalence, incidence and impact across Latin American countries, China and India, using a validated and common methodology. However, given that this was a population cohort, the scope of the research was much broader than this—entailing a comprehensive enquiry into health (common and burdensome chronic diseases, disability and health service utilization), and social aspects of ageing (socioeconomic status, social protection, needs for care and care arrangements).

Fundamental methodological issues, in particular the development, calibration and validation of culture- and education-fair dementia diagnosis, and care arrangements for care-dependent older people, were addressed in pilot investigations in 26 centres from 16 low- and middle-income countries in Latin America and the Caribbean, Africa, India, Russia, China and South East Asia (1999–2001).^2–5^ The protocols for the 10/66 DRG baseline surveys and incidence phase surveys have already been described in detail in a previous publication.^6^ The purpose of this paper is to describe in more detail the resources created through these completed surveys, together with findings from the research completed to date and further plans for development of this resource.

## Where is it located, who set it up and how has it been funded?

The 10/66 DRG is coordinated from London, within the Centre for Global Mental Health at King’s College London, with a network of centres each led by a local principal investigator. The 10/66 DRG’s research has been funded by the Wellcome Trust Health Consequences of Population Change Programme (GR066133—Prevalence phase in Cuba and Brazil; GR08002—Incidence phase in Peru, Mexico, Cuba, Dominican Republic, Venezuela and China), World Health Organization (India, Dominican Republic and China), the US Alzheimer’s Association (IIRG–04–1286—Peru and Mexico), FONACIT/ CDCH/ UCV (Venezuela), and Puerto Rico Legislature (data collection in Puerto Rico) and Pfizer Co., USA (blood sample collection in Puerto Rico). The new cohort is funded by a European Research Council Advanced Grant (340755). The Rockefeller Foundation supported our dissemination strategy meeting at their Bellagio Centre. Alzheimer Disease International (ADI) has provided support for networking and infrastructure.

## Who is in the cohort?

**Figure 1:**
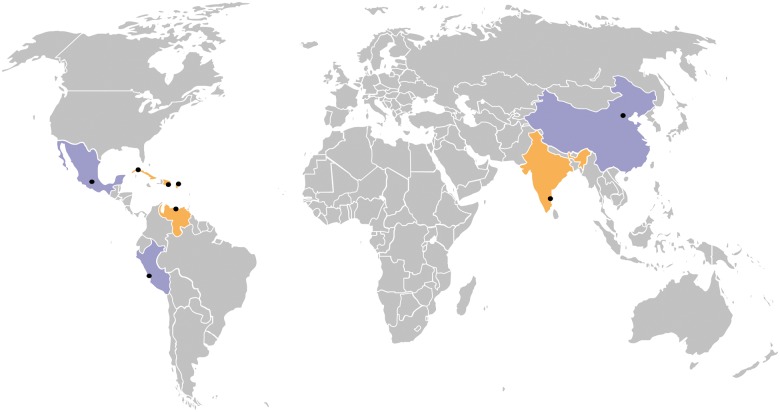
Distribution of the 10/66 centres. Countries in purple contain both rural and urban centres, whereas countries in orange only have urban centres. The black dots represent the catchment areas within each country. The centres are the following: China (Beijing and Daxing), Cuba (Havana/Matanzas), Dominican Republic (Santo Domingo), India (Chennai), Mexico (Mexico City and Morelos/Hidalgo), Peru (Lima and Canete), Puerto Rico (San Juan) and Venezuela (Caracas).

**Figure 2:**
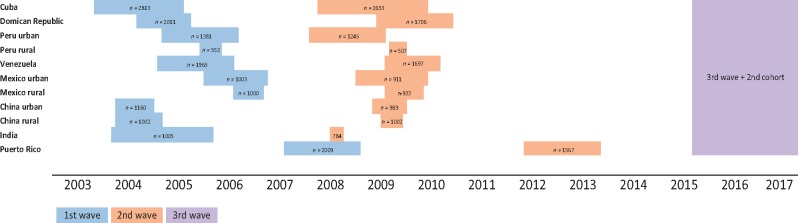
Cohort diagram of the baseline and follow-up surveys; 2^nd^ wave numbers refer to number of people with a determined vital status.

The 10/66 cohort is a population cohort comprising in principle all older residents aged 65 years and over, living in 11 geographically defined urban and rural catchment area sites in eight low- and middle-income countries. The selection of catchment areas for the baseline phase of the survey was purposive, based upon their accessibility, their use in the past as field sites for community or population research and the existence of or potential for development of good relationships between the local research groups and community stakeholders. Urban sites were selected to comprise mixed or mainly lower socioeconomic status households; exclusively high-income or professional districts were excluded. Urban sites were located in Cuba (one catchment area comprising sites is Havana and Matanzas), Dominican Republic (Santo Domingo), Puerto Rico (Bayamon), Venezuela (Caracas), Peru (Lima), Mexico (Mexico City), China (Xicheng, Beijing province) and India (Chennai). Rural sites, selected to be remote from major population centres, with low-density population and with agriculture and related trades as the main local employment, were located in Peru (Canete Province), Mexico (Morelos State) and China (Daxing, Beijing Province). The centre and site characteristics are summarized in Figure 1. The baseline phase was conducted for all centres between 2004 and 2006, with the exception of Puerto Rico where baseline data were collected between 2007 and 2010 (Figure 2). Mapping of the catchment areas was carried out within specified boundaries, and households were allocated household identification numbers. Enumeration was carried out by door-knocking all households in the catchment area to identify potentially eligible participants (those aged 65 years or over on a census date) who were then allocated participant identification numbers. These are linked to names and addresses in secure databases held in London. Participants’ ages were confirmed during the interview. Information about the age and sex of all other co-residents was also recorded. After verifying eligibility, written consent was obtained from participants or next of kin if the individual lacked capacity. Oral consent, witnessed in writing by someone literate, was taken from illiterate participants. An overall sample of 2000 per country would allow estimation of a typical dementia prevalence of 4.5% with a precision of ± 0.9%, and rural and urban samples of 1000 each would allow estimation of the same prevalence with a precision of ± 1.2%. A sample size of around 2000 individuals for each country was achieved and the response rate was excellent in most catchment areas, with a range of 72 % to 98% by site, and an average across sites of 86% (Table 1).

**Table 1.** Baseline sample socio-demographic characteristics and response rate by study centre

	Cuba (%)	Dominican Republic (%)	Puerto Rico (%)	Peru urban (%)	Peru rural (%)	Venezuela (%)	Mexico urban (%)	Mexico rural (%)	China urban (%)	China rural (%)	India urban (%)
Participants (*n*)	2813	2011	2009	1381	552	1965	1003	1000	1160	1002	1005
Response rate (%)	94	95	93	80	88	80	84	86	74	96	72
Women	1836 (65.3)	1325 (66.0)	1347 (67.3)	888 (64.3)	295 (53.4)	1226 (63.5)	666 (66.40)	602 (60.20)	661 (57.0)	556 (55.49)	571 (57.7)
**Age (years)**											
65–69	715 (25.5)	533 (26.5)	414 (20.6)	375 (27.2)	179 (32.4)	839 (42.8)	245 (24.4)	299 (29.9)	316 (27.2)	383 (38.2)	415 (41.5)
70–74	747 (26.6)	520 (25.9)	456 (22.7)	352 (25.5)	141 (25.5)	469 (23.9)	329 (32.8)	252 (25.2)	362 (31.2)	296 (29.5)	318 (31.8)
75–79	618 (22.0)	397 (19.7)	483 (24.0)	298 (21.6)	101 (18.3)	345 (17.6)	205 (20.5)	221 (22.1)	254 (21.9)	202 (20.2)	144 (14.4)
≥ 80	726 (25.9)	561 (27.9)	656 (32.6)	355 (25.7)	131 (23.7)	308 (15.7)	223 (22.3)	228 (22.8)	228 (19.7)	121 (12.1)	124 (12.4)
**Marital status**											
Never married	262 (9.3)	139 (7.0)	123 (6.1)	145 (10.6)	68 (12.3)	189 (9.8)	63 (6.3)	42 (4.2)	3 (0.3)	22 (2.2)	21 (2.1)
Married/cohabiting	1199 (42.8)	586 (29.4)	967 (48.3)	784 (57.2)	308 (55.9)	921 (48.0)	470 (46.9)	538 (53.8)	829 (71.5)	585 (58.4)	523 (52.2)
Widowed	896 (31.9)	806 (40.4)	672 (33.6)	367 (26.8)	157 (28.5)	549 (28.6)	395 (39.4)	371 (37.1)	326 (28.1)	394 (39.3)	426 (42.5)
Divorced/separated	448 (16.0)	465 (23.3)	240 (12.0)	75 (5.5)	18 (3.3)	261 (13.6)	75 (7.5)	48 (4.8)	2 (0.2)	1 (0.1)	32 (3.2)
**Education**											
None	73 (2.6)	392 (19.7)	72 (3.6)	37 (2.7)	84 (15.4)	156 (8.1)	227 (22.6)	327 (32.7)	232 (20.0)	579 (57.8)	428 (42.7)
Minimal	619 (22.1)	1022 (51.3)	389 (19.4)	90 (6.5)	141 (25.9)	445 (23.1)	354 (35.3)	510 (51.0)	153 (13.2)	114 (11.4)	234 (23.3)
Completed primary	937 (33.4)	370 (18.6)	415 (20.7)	460 (33.5)	267 (49.1)	965 (50.1)	229 (22.8)	122 (12.2)	303 (26.1)	259 (25.8)	212 (21.1)
Completed secondary	705 (25.1)	135 (6.8)	713 (35.5)	481 (35.0)	36 (6.6)	266 (13.8)	99 (9.9)	25 (2.5)	335 (28.9)	45 (4.5)	87 (8.7)
Tertiary	471 (17.0)	73 (3.7)	410 (20.4)	305 (22.2)	16 (2.9)	93 (4.8)	94 (9.4)	16 (1.6)	137 (11.8)	5 (0.5)	42 (4.2)
**Number of assets**											
0–2	27 (1.0)	136 (6.8)	4 (0.2)	5 (0.4)	38 (6.9)	39 (2.0)	13 (1.3)	213 (21.3)	0	15 (1.5)	132 (13.2)
3–5	957 (34.1)	951 (47.4)	38 (1.9)	61 (4.4)	343 (62.1)	9 (0.5)	150 (15.0)	518 (51.8)	604 (52.1)	374 (37.3)	620 (61.9)
6–7	1821 (64.9)	919 (45.8)	1967 (97.9)	1315 (95.2)	171 (31.0)	1917 (97.6)	840 (83.7)	269 (26.9)	555 (47.9)	613 (61.2)	249 (24.9)
**Occupation**											
Professional	1027 (38.8)	333 (16.6)	69 (8.3)	613 (45.5)	51 (9.3)	609 (34.0)	208 (20.7)	30 (3.0)	624 (54.1)	40 (4.0)	139 (14.9)
Trade	378 (14.3)	264 (13.2)	188 (22.7)	266 (19.7)	37 (6.7)	429 (23.9)	171 (17.0)	50 (5.0)	56 (4.9)	2 (0.2)	131 (14.1)
Semi-skilled	759 (28.7)	751 (37.5)	62 (7.5)	378 (28.0)	104 (18.9)	662 (36.9)	318 (31.7)	300 (30.0)	374 (32.4)	16 (1.6)	324 (34.8)
Labourer	469 (17.7)	640 (31.9)	3 (0.4)	22 (1.6)	29 (5.3)	77 (4.3)	257 (25.6)	440 (44.0)	96 (8.3)	16 (1.6)	224 (24.1)
Agricultural worker	203 (7.7)	15 (0.7)	507 (61.7)	69 (5.1)	330 (59.9)	16 (0.9)	49 (4.9)	180 (18.0)	3 (0.3)	928 (92.6)	112 (12.0)
**Food insecurity**											
Yes	137 (4.9)	240 (12.1)	32 (1.6)	63 (4.6)	74 (13.5)	111 (6.0)	39 (3.9)	85 (8.6)	0 (0)	12 (1.2)	207 (20.8)
**Living arrangements**											
Alone	250 (8.8)	254 (12.6)	472 (23.5)	45 (3.3)	44 (8.0)	61 (3.1)	106 (10.6)	112 (11.2)	54 (4.7)	49 (4.9)	44 (4.4)
With spouse	426 (15.1)	135 (6.7)	666 (33.2)	126 (9.1)	59 (10.7)	135 (6.9)	151 (15.1)	156 (15.6)	415 (35.8)	140 (14.0)	194 (19.4)
With adult children	1340 (47.6)	963 (47.9)	288 (14.3)	890 (64.4)	326 (59.1)	1578 (80.3)	565 (56.3)	523 (52.3)	446 (38.4)	679 (67.8)	719 (71.5)
**Smoking status**											
Never	1612 (54.9)	1049 (52.2)	1454 (72.6)	1119 (81.4)	482 (87.5)	1061 (55.8)	648 (64.6)	729 (72.9)	875 (75.4)	666 (66.5)	730 (73.2)
Ex-smoker	759 (25.8)	711 (35.4)	444 (22.2)	201 (14.6)	55 (10.0)	624 (32.8)	246 (24.5)	200 (20.0)	92 (7.9)	31 (3.1)	86 (8.6)
Current	565 (19.2)	249 (12.4)	104 (5.2)	54 (3.9)	14 (2.5)	215 (11.3)	109 (10.9)	71 (7.1)	193 (16.6)	305 (30.4)	181 (18.2)

## The completed cohort resource

The cohort at baseline, with respect to vital status ascertained through to March 2014, comprised 15 901 participants at risk (Table 1; and Supplementary Table 1, available as Supplementary data at *IJE* online). The median follow-up period ranged from 2.8 to 5.0 years by site, with a total of 53 872 person-years of observation. The vital status of 13 936 participants (87.7%) was determined, with 2602 deaths occurring during the follow-up period, for which 2436 verbal autopsy interviews were completed. The proportion deceased at follow-up was higher in China, Dominican Republic and Cuba than in other countries (in part a function of the longer follow-up interval in those sites). With respect to the incidence of dementia (further excluding India, where dementia-free participants were not followed up), 14 896 participants were interviewed at baseline, 13 483 free of dementia; 9322 (69.1%) were re-interviewed, contributing 42 698 person-years of follow-up.

## How often have they been followed up?

Participants of the baseline assessment were traced and followed up between 2007 and 2010 in the China, India (mortality only), Cuba, Dominican Republic, Mexico, Peru and Venezuela sites, and between 2012 and 2013 in Puerto Rico. In India, all those with mild cognitive impairment, ‘cognitive impairment no dementia’ (CIND) or dementia at baseline completed the full incidence phase protocol, to determine the predictive validity of baseline dementia diagnosis.^7^ A mortality sweep was carried out on the full baseline cohort to determine vital status, date of death of those deceased and a verbal autopsy on those deceased.^8^

Subsequent to the baseline and incidence waves, the 10/66 INDEP sub-study has been completed,^9^ a nested study of households in Peru, Mexico and China, characteriszd as ‘incident care’, ‘chronic care’ or ‘no care’, depending upon the needs for care of older residents. This focuses on the economic and social functioning of the household as a whole. A third full wave of assessment using an extended form of the basic 10/66 survey has recently been planned and funded for Cuba, Dominican Republic, Puerto Rico, Mexico, Peru, Venezuela and China, which will take place approximately 10 years on from the original baseline surveys (2015–16).This will be a new prevalence sweep, with renewed -door-knocking of the original catchment areas to generate a new representative prevalence sample of all those aged 65 years and over, including those that have aged to 65 years or over since the first prevalence wave, and in-migrants. Participants of the original baseline survey will be traced and have their vital status ascertained, and be re-interviewed where possible even if migrated out of the area.

## What has been measured?

The same cross-culturally validated assessment was carried out across each centre, during the baseline and follow-up phase of the study. All participants underwent a comprehensive interview, including a structured interview, a physical examination and an informant interview. Key informants were selected by interviewers on the basis of who knew the old person best and could give the clearest and most detailed account of their current circumstances. Co-residents and family members were prioritized unless others were clearly better qualified. The main criterion for selection in case of several co-resident family members was time spent with the older person. In cases where the older person needed care, then the main caregiver was selected. However, if the main caregiver was paid, the main organizational caregiver was selected instead.

Each full assessment, which lasted between 2 and 3 h, was translated, back-translated and adapted as necessary into the different languages for each centre (Table 2).

**Table 2.** Measurements in the different waves of studies. WHODAS (World Health Organization Disability Assessment Schedule), DEMQOL (Dementia Quality Of Life Questionnaire), CSI’D’ (Community Screening Instrument for Dementia), HAS (History and Aetiology Schedule), NPI (Neuropsychiatric Inventory), GMS (Geriatric Mental State Examination)

	Baseline	Follow-up	3rd wave/refreshment cohort
**Household assessment**	x	x	x
Age ascertainment	x		x
Household information	x	x	x
Number of assets	x	x	x
**Participant interview**	x	x	x
Early-life events	x	x	x
Current circumstances	x	x	x
Social network	x	x	x
Socioeconomic status	x	x	x
Health (including pain and impairments)	x	x	x
Disability (WHODAS-II) and dependence	x	x	x
Reproductive health	x	x	x
Behaviour and lifestyles	x	x	x
Use of health services	x	x	x
Quality of life (DEMQOL)		x	x
**Cognitive functioning**			
CSI’D’	x	x	x
10-word list-learning test	x	x	x
Mental health (GMS – version B3)	x	x	x
**Clinical examination**			
Neurological assessment (NEUROEX)	x	x	x
Physical assessment (anthropometry, pulse/blood pressure)	x	x	x
Stroke assessment		x	
Advanced frailty assessment			x
**Biological assessments**			
Haematological tests, full blood count (haemoglobin, haematocrit, differential, MCV, MCH, MCHC)	x	x	x
	(some centres)	(some centres)	
Biochemical tests (fasting glucose, fasting total cholesterol and sub-fractions, triglyceride, albumin, total protein)	x	x	x
	(some centres)	(some centres)	
Genotyping (ApoE)	x	x	x
	(some centres)	(some centres)	
Metabolic syndrome according to NCEP-ATP III criteria	x	x	x
	(some centres)	(some centres)	
Age-related decline biomarkers (cytokines, telomeres, CRP, testosterone, SHBG)			x
			(nested-cohort)
**a) Informant interview**			
Background information on informant	x	x	x
Caregiver questionnaire	x	x	x
CSI’D’ informant section	x	x	x
HAS-D	x	x	x
Behavioural and Psychological Symptoms of Dementia (NPI)	x	x	x
Participants background info^a^	x	x	x
DEMQOL		**x**	**x**
Verbal autopsy		**x**	**x**

^a^Administered when the participant is too demented or otherwise unable to answer the questions reliably.

### Socio-demographics

Information on age, sex, marital status, level of education (none; some, but did not complete primary; completed primary; completed secondary; completed tertiary or further education), household assets and household composition was assessed by a standard socio-demographic questionnaire.

### Health

For some conditions, health status was assessed using self-reported diagnoses, in response to the question ‘has a doctor ever told you that you suffered from’: stroke, diabetes, hypertension, heart disease (and hyperlipidaemia, at follow-up only), TB, malaria or cysticercosis, and treatments for these conditions. Directly assessed diagnoses included: (i) Dementia, ascertained according to the cross-culturally validated 10/66 dementia diagnosis algorithm^3^ and the DSM-IV dementia criterion^10^ after cognitive testing, clinical and informant interview; (ii) Depression according to ICD-10 criteria and EURO-D scale scores, and syndromal levels of anxiety and psychosis ascertained using the structured Geriatric Mental State clinical interview (GMS);^11–13^ (iii) Hypertension according to European Society of Hypertension criteria (systolic blood pressure > = 140 mmHg and/or diastolic blood pressure > = 95 mmHg, and/or a positive answer to the question ‘have you ever been told by a doctor that you have hypertension?’); (iv) Chronic Obstructive Pulmonary Disease (COPD) diagnosed in those who responded ‘yes’ to the question ‘do you usually cough up phlegm from your chest first thing in the morning?’ and whose answer to the question ‘for how many months of the year does this usually happen?’ was 3 months or more.

Self-rated overall health and physical impairments (including eyesight problems; stomach or intestine problems; arthritis or rheumatism; heart problems; hearing difficulties or deafness etc.) were also assessed. Impairments were rated as present if they interfered with activities ‘a little’ or ‘a lot’, as opposed to ‘not at all’.^14^ Women’s reproductive history (menarche, menopause and parity) was also assessed. The informant rated the presence and severity of any behavioural and psychological symptoms (Neuropsychiatric Inventory Questionnaire -NPI-Q).^15^

Finally, a physical examination was carried out comprising pulse and blood pressure, height, leg length, skull, arm, waist and hip circumference and a structured neurological examination (NEUROEX).^6^ At follow-up only, weight and calf circumference were also assessed.

### Impacts of health

1. Disability. Disability was measured using the 12-item World Health Organization Disability Assessment Schedule version 2.0 (WHODAS 2.0). The WHODAS 2.0 has high internal consistency, moderate to good test-retest reliability and good concurrent validity in many different chronic disease clinical populations.^16–18^
2. Dependence. The interviewer administered open-ended questions to the key informant, to ascertain needs for care. The interviewer then coded whether the participant required no care, care some of the time or care much of the time. This coding was based upon the interviewer’s perception of needs for care, independently of whether these were routinely met. Conditionally upon the presence of needs for care, we further assessed: (i) Practical impact—contact time between caregiver and cared-for person^19^ and time spent by the caregiver in the past 24 h in specific caregiving activities;^20^ (ii) Caregiver perceived strain—the Zarit Burden Interview (ZBI)^21^^,^^22^ with 22 items that assess the caregiver’s appraisal of the impact their involvement has had on their lives.
3. Health service utilization was assessed using the Client Service Receipt Inventory,^23^ a comprehensive assessment of direct and indirect economic costs for mental health services, adapted for use in the developing world.^24^ Help-seeking, specifically for symptoms and signs of dementia, was assessed at follow-up only.

### Risk exposures

Specific dementia risk factors (e.g. head injury with loss of consciousness, family history of dementia) and broader lifestyle and cardiovascular risk factors including alcohol use (volume and frequency currently and before the age of 60), lifetime smoking (including pack-year calculation) and diet and exercise levels now and in earlier life, were also assessed.

### Biological samples

Fasting blood samples were collected in a subset of seven Latin American sites (Cuba, Dominican Republic, Venezuela, Puerto Rico, urban Peru, and urban and rural Mexico), for which we are also able to report the prevalence of undiagnosed diabetes and the extent of control among diagnosed cases. We collected fluoride oxalate, EDTA and clot samples. Haematological and biochemical analyses were carried out in local laboratories. DNA was extracted to create a resource for genotyping. The range of assays carried out varied among sites, depending on feasibility and funding (see Table 2; and Supplementary Table2, available as Supplementary data at *IJE* online).

Overall, 9178 blood samples were collected. By site, the numbers and proportions providing samples were: Cuba (2355, 80.4% of those participating in the survey), Dominican Republic (1483, 73.8%), Puerto Rico (1584, 78.8%), Venezuela (1284, 65.3%), urban Peru (755, 54.7%), urban Mexico (822, 82.0%) and rural Mexico (895, 89.5%). There were few differences in baseline characteristics of those who did and did not provide samples (Supplementary Table 3, available as Supplementary data at *IJE* online), and those differences were generally of small effect, other than in the urban Peru site where the more affluent and better educated, but also those with more physical impairments, were more likely to give blood samples.

The new third-wave prevalence survey will include an extended assessment of health status, including spirometry, body mass index (BMI), visual acuity, grip strength and hearing impairment. Moreover, a nested cohort of 300 individuals (150 with a high risk of incident dependence and 150 without) will be identified and extensive laboratory testing of frailty biomarkers carried out. This sub-group will be followed up 18 months later to re-assess vital status, needs for care, disability, cognitive function and significant health life events in the intervening period.

## What has it found? Key findings and publications

Evidence on the construct and predictive validity of key measures has been further strengthened. Norms for the cognitive tests indicate effects of age and education, and a modest effect of culture upon the cognitive tests (10-word delayed recall and CSI-D COGSCORE) that form the core of our 10/66 dementia diagnosis.^25^ Our confidence in the validity of the 10/66 dementia diagnosis has been bolstered by the demonstration, in Cuba, that it agreed better with Cuban clinician diagnoses than did the DSM-IV computerzsed algorithm, which missed many recent-onset and mild cases.^10^ Across the cohort, levels of disability were similar for 10/66 dementia cases regardless of whether they were confirmed as cases by the DSM-IV dementia algorithm.^26^ Crucially, in urban India where the disparity between the prevalence of 10/66 dementia and DSM-IV dementia was greatest, those with 10/66 dementia had a markedly elevated mortality rate, and survivors showed clear evidence of clinical progression and increased needs for care. Only one ‘case’ had unambiguously improved. Cognitive function had deteriorated and disability increased to a much greater extent than among those with CIND. Hence, the strong predictive validity of the 10/66 dementia diagnosis is consistent with a lack of sensitivity of the DSM-IV criteria to mild-to-moderate cases, which may underestimate prevalence in less developed regions. Regarding the WHODAS 2.0 disability assessment scale, in the 10/66 DRG population-based survey samples strong internal consistency and high factor loadings for the one-factor solution supported unidimensionality, and the WHODAS 2.0 was found to be a ‘strong’ Mokken scale in all sites.^27^

Morbidity in the baseline surveys of the cohort has been described in detail, with publications on the prevalence of dementia,^26^ mild cognitive impairment,^28^ mental disorder,^29–32^ sleep disorder,^33^ hypertension,^34^ stroke,^35^ anaemia,^36^ head injury^37^ and dependence^38^ (Table 3). Prevalence of most chronic disorders, including dementia, is similar to that in high-income countries for urban settings in Latin America and China, and somewhat lower in rural settings and in India. Detailed country reports delineate the impact of population ageing and the epidemiological transition on patterns of chronic disease morbidity and needs for care in Cuba,^39^ Dominican Republic^40^ and China.^41^ The independent impact of different chronic diseases and frailty^42^ on disability,^43^ dependence,^38^ co-resident psychological morbidity,^44^ service utilization^24^ and costs,^45^ indicating a predominant contribution of disorders of the brain and mind (dementia, stroke and depression) to disability, dependence and costs, but an inverse association between dementia and healthcare service utilization.

**Table 3.** Morbidity at baseline across sites, *n* (%)

	Cuba	Dominican Republic	Puerto Rico	Peru urban	Peru rural	Venezuela	Mexico urban	Mexico rural	China urban	China rural	India urban
Dementia^a^	292 (10.4)	235 (11.7)	233 (11.7)	129 (9.34)	36 (6.5)	140 (7.1)	86 (8.6)	85 (8.5)	81 (7.0)	556 (5.6)	75 (7.5)
Mild cognitive impairment^b^	42 (1.5)	26 (1.3)	68 (3.4)	36 (2.6)	18 (3.3)	22 (1.1)	27 (2.7)	32 (3.2)	5 (0.4)	12 (1.2)	33 (3.3)
Stroke^c^	216 (7.7)	175 (8.7)	168 (8.4)	112 (8.2)	20 (3.6)	135 (7.0)	67 (6.7)	74 (7.4)	109 (9.4)	18 (1.8)	20 (2.0)
Hypertension^d^	1624 (57.9)	968 (48.6)	518 (32.1)	209 (15.2)	37 (6.7)	714 (46.5)	423 (42.2)	371 (37.2)	489 (42.4)	557 (55.6)	608 (60.7)
Alcohol problems											
Early life	212 (7.6)	605 (30.4)	132 (6.6)	18 (1.3)	17 (3.1)	88 (7.4)	80 (8.1)	112 (11.3)	26 (2.2)	73 (7.3)	4 (0.4)
Current	103 (3.7)	234 (11.7)	28 (1.4)	3 (0.2)	5 (0.9)	17 (1.5)	9 (0.9)	11 (1.1)	17 (1.5)	42 (4.2)	1 (0.1)
Needs for Care^f^	157 (6.4)	143 (7.1)	182 (9.1)	75 (5.4)	10 (1.8)	98 (5.0)	56 (5.6)	30 (3.0)	119 (10.3)	30 (3.0)	14 (1.4)
Depression^g^	142 (5.1)	278 (13.8)	47 (2.3)	87 (6.3)	16 (2.9)	107 (5.5)	47 (4.7)	45 (4.5)	3 (0.3)	7 (0.7)	39 (3.9)

^a^10/66 education-adjusted dementia diagnosis.

^b^Petersen criteria amnesic MCI.

^c^Self reported stroke.

^d^Meeting ISH hypertension criteria (≥ 14 mmHg systolic and/or ≥ 90 mmHg diastolic).

^e^Hazardous drinker.

^f^Needing much care most of the time.

^g^ICD-10 Depression.

The incidence of 10/66 dementia ranged from 18.2 to 30.4 per 1000 person-years, similar or higher than the incidence of dementia reported for high-income countries. Incidence of 10/66 dementia was 1.4–2.7 times higher than that for DSM-IV dementia (15.7 and 9.9 per 1000 person-years, respectively).^46^ Mortality hazards ratios for dementia ranged from 1.56 to 5.69 by site.^46^ Education [hazard ratio (HR): 0.89, 95% confidence interval (CI): 0.81–0.97], and male sex (0.72, 0.61–0.84) were inversely associated, and older age [risk ratio (RR) per 5-year band 1.67, 1.56–1.79] were all associated with incident dementia. Literacy, motor sequencing and verbal fluency all protected against dementia onset, independent of education, providing support for the cognitive reserve hypothesis.

Crude mortality rates varied from 27.3 to 70.0/1000 person-years, a 3-fold variation persisting after standardization for demographic and economic factors.^47^

A full list of findings and publications can be found on the study website [http://www.alz.co.uk/1066/1066_publications.php] and in Supplementary Table 4 (available as Supplementary data at *IJE* online).

## What are the main strengths and weaknesses?

One of the main strengths of this study was the use of a one-phase design to estimate the prevalence, incidence, determinants and consequences of a comprehensive range of chronic conditions, with a particularly robust dementia assessment procedure specifically developed and validated for use in LMIC. The same standardized protocol, which included validated measurements and diagnostic algorithms, was used in each site, permitting comparison of estimates across diverse settings and aiding the interpretation of observed variations. The relatively large sample size also allows quite precise estimations of effect sizes, in particular with meta-analysed pooled estimates. Response rates were also high, and the number of missing values relatively low. Although the use of catchment areas increased the response rates, this could have affected the generalizability of the findings beyond the specific study sites.

## Can I get hold of the data? Where can I find out more?

The 10/66 cohort is an open-access database, and we would encourage external investigators to consider applying to use the data for secondary analyses, in order to maximize the scientific output from the data. All the information on how to access the 10/66 public data archive, with a list of current proposals and papers currently under preparation, can be found on our website: [www.alz.co.uk/1066/].

## Supplementary Data

Supplementary data are available at *IJE* online.

## Funding

This work was supported by the Wellcome Trust (GR066133/ GR080002), the European Research Council (340755), US Alzheimer’s Association, WHO, FONDACIT (Venezuela) and the Puerto Rico State Government, and the Medical Research Council (MR/K021907/1 to A.M.P.)

**Conflict of interest:** None.

## Supplementary Material

Supplementary Data
